# Novel nano‐hydroxyapatite coating of additively manufactured three‐dimensional porous implants improves bone ingrowth and initial fixation

**DOI:** 10.1002/jbm.b.35165

**Published:** 2022-09-28

**Authors:** Ryota Watanabe, Hiroyuki Takahashi, Aira Matsugaki, Toru Uemukai, Yasumichi Kogai, Takashi Imagama, Kiminori Yukata, Takayoshi Nakano, Takashi Sakai

**Affiliations:** ^1^ R&D Center Teijin Nakashima Medical Co., Ltd. Okayama Okayama Japan; ^2^ Division of Materials and Manufacturing Science, Graduate School of Engineering Osaka University Osaka Japan; ^3^ R&D Center SofSera Co. Ltd. Ibaraki Osaka Japan; ^4^ Department of Orthopedic Surgery Yamaguchi University Graduate School of Medicine Ube Yamaguchi Japan

**Keywords:** 3D porous acetabular cup, bone implant fixation, bone ingrowth, electron beam melting, nano‐hydroxyapatite coating

## Abstract

Electron beam melting (EBM) has been used to fabricate three‐dimensional (3D) porous Ti‐6Al‐4V surfaces for acetabular cups in total hip arthroplasty. However, there are radiographic concerns regarding poor implant fixation and bone ingrowth around electron beam melted (EBMed) 3D porous cups. We hypothesize that nano‐hydroxyapatite (nHA) coating can promote bone ingrowth and thus decrease the occurrence of radiolucent lines around EBMed 3D porous cups. This study aimed to investigate the effect of a novel nHA coating on the biological performance of EBMed 3D porous implants in a beagle transcortical model. Low‐porosity (control) and high‐porosity 3D porous Ti‐6Al‐4V implants were manufactured using EBM. Half of the high‐porosity implants were coated with nHA without clogging the 3D pores. Implants were inserted into the femoral diaphysis of the beagles. The beagles were euthanized at 4, 8, and 12 weeks postoperatively, and push‐out testing was performed. Bone ingrowth was evaluated by histological analysis. Although the increase in porosity alone had no effect on biological behavior, the addition of nHA to high‐porosity 3D implants significantly improved early bone fixation and bone ingrowth into the deep region of porous structures compared to low‐porosity implants. This is the first report of a novel nHA coating that improved bone ingrowth into the deeper regions of 3D porous implants, which can prevent the occurrence of radiolucent lines around EBMed 3D porous cups.

## 1 INTRODUCTION

Joint disorders, such as osteoarthritis, cause pain and reduce quality of life. Total hip arthroplasty (THA) is one of the most successful orthopedic procedures for the treatment of joint diseases, and the number of patients using artificial hip joints is expected to increase in the future. The long‐term success of THA is dependent on the biological fixation of the implant in the bone tissue. A variety of surface treatments, such as grit blasting, fiber mesh, hydroxyapatite (HA) coating, and trabecular‐like structures, have been developed to improve bone‐implant fixation.^1^, ^2^, ^3^, ^4^ Although good clinical outcomes have been obtained with surface treatments, aseptic loosening due to a lack of bone ongrowth or ingrowth is one of the main causes of revision surgery.^5^

To date, additive manufacturing (AM) techniques have attracted considerable attention in THA because they enable the fabrication of complex three‐dimensional (3D) porous structures with high pore size and porosity,^6^, ^7^, ^8^, ^9^ which has been shown to have a significant effect on bone ingrowth, bone‐implant fixation, and biological behaviors.^10^, ^11^, ^12^ Various porous structures have been applied to acetabular cups in artificial hip joints^13^ with excellent clinical results.^14^, ^15^ However, some studies have reported a high rate of radiolucent lines around 3D porous cups fabricated by AM techniques, which indicate poor osseointegration and bone ingrowth.^16^, ^17^ Therefore, bioactive coatings that induce early bone formation into 3D porous structures are required to prevent radiolucent lines.

HA has been widely used as a bioactive coating material in artificial joints for several decades.^18^, ^19^, ^20^ A recent study revealed that the frequency of radiolucent lines around plasma‐sprayed HA‐coated cups was lower than that in 3D porous cups additively manufactured by electron beam melting,^16^ indicating that HA coating is an effective way to promote bone formation. However, micro‐sized HA is difficult to coat onto porous structures by plasma spraying because HA quickly clogs the pores. We focused on smaller and highly dispersible hydroxyapatite particles, that is, nano‐hydroxyapatite (nHA), to coat electron‐beam melted (EBMed) 3D porous structures without clogging the pores. Although nHA coating has been reported to enhance bone formation around implant surfaces in animal models,^21^, ^22^ its effect on EBMed 3D porous Ti‐6Al‐4V implants has not been investigated.

The aim of this study was to investigate the effect of a novel nHA coating on the biological behavior of EBMed 3D porous implants using a beagle transcortical model, which has similar bone structure and composition to human bones.^23^ The influence of increased porosity was also evaluated. Mechanical stability and bone ingrowth were evaluated at 4, 8, and 12 weeks after implantation.

## 2 MATERIALS AND METHODS

### 2.1 Sample preparation

**FIGURE 1:**
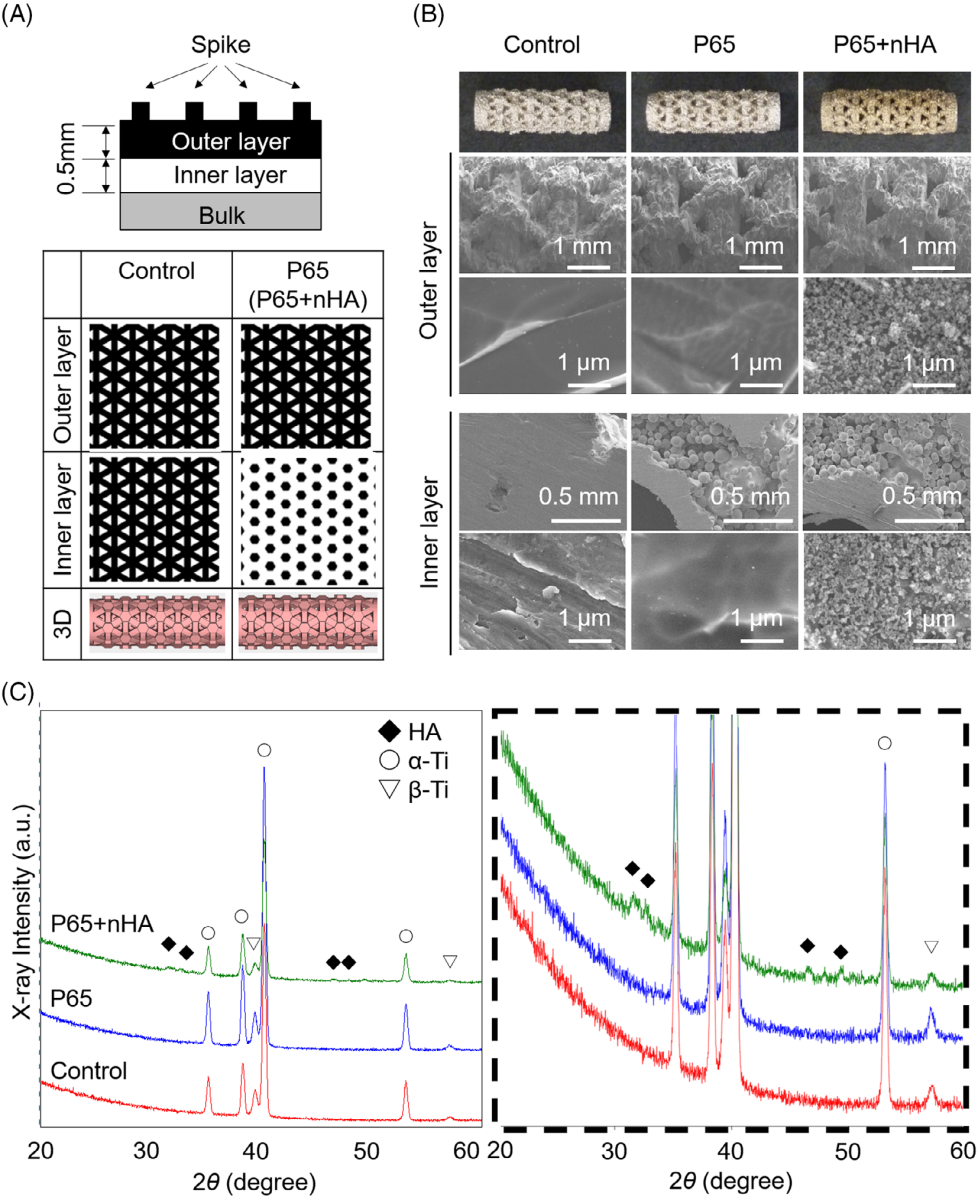
(A) Schematic illustrations of the porous structures of each implant. (B) field‐emission scanning electron microscopy images of outer and inner surface of each implant at low and high magnifications. (C) X‐ray diffraction (XRD) patterns for each implant (left). The magnified patterns of XRD analysis for all implants shown inside the dashed box (right)

A gas‐atomized Ti‐6mass%Al‐4mass%V (Ti‐6Al‐4V) extra low interstitial alloy powder was used in this study. Two types of cylindrical porous Ti‐6Al‐4V implants (4.5 mm in diameter and 13 mm length) were additively manufactured using electron beam melting (Model A2X; Arcam); one had 38% porosity (control) and the other had 65% (P65). The inner layer of the P65 structures had a higher porosity than the control, while the outer layer had the same porosity as the control (Figure 1A). The pores were designed as equilateral triangle structure. The pore size of 650 μm was defined as the diameter of the circle with the equivalent area and designed using the computer aided design model (CAD) data.

Sphere‐shaped hydroxyapatite nanoparticles (SofSera, Tokyo, Japan) with a mean particle size of 40 nm were prepared, referring to the previous reported method.^24^ Briefly, original HA particles were prepared by an emulsion system. nHA was obtained by calcination of the original HA particles with an anti‐sintering process. Half of the implants with 65% porosity were coated with nano‐hydroxyapatite (P65 + nHA). The implants were then heated at 300°C for 30 min in air. After heat treatment, the implants were immersed in a mixture of toluene (FUJIFILM Wako Pure Chemical Corporation, Osaka, Japan) and a silane coupling agent (Tokyo Chemical Industry Co., Ltd., Tokyo, Japan) and placed in an oven at 60°C for 60 min to silanize the implant. The implants were then dried under reduced pressure at room temperature. The silanized implants were soaked in a nHA suspension in ethanol (FUJIFILM Wako Pure Chemical) at 60°C for 60 min to adsorb the nHA particles. Heat treatment under reduced pressure was performed at 120°C for at least 60 min to stabilize the nHA particles on the implants. All implants were ultrasonically washed with acetone and distilled water and then sterilized by gamma‐ray irradiation.

The surfaces of the implants were observed using field‐emission scanning electron microscopy (FE‐SEM; S‐4800, Hitachi, Tokyo, Japan) at an accelerated voltage of 5 kV. nHA was confirmed to be coated on the surface of both outer and inner pores of P65 + nHA group, which did not clog the pores of the EBMed 3D porous implants (Figure 1B). X‐ray diffraction (XRD; SmartLab, Rigaku, Osaka, Japan) analysis was performed to determine the crystalline phase of the implants with Cu‐Kα radiation (45 kV and 200 mA) between 20° and 60° (2*θ*). In XRD patterns, the diffraction peaks corresponding to titanium and HA were detected in P65 + nHA (Figure 1C).

### 2.2 Surgical procedure

All animal experiments were approved by the Institutional Animal Care and Use Committee of Hamri Co. Ltd. (20‐H033). Nine male beagles aging 12–15 months and weighing 8.90–10.50 kg were allocated into three groups with similar average body weights. Beagles were anesthetized using a combination of 20 mg/kg ketamine hydrochloride (Daiichi Sankyo Propharma Co., Ltd., Tokyo, Japan) and 2 mg/kg xylazine (Bayer Yakuhin Ltd., Osaka, Japan). Four drill holes (4.5 mm diameter and 10 mm depth) were made laterally in the diaphysis of each femur using a hand drill. The implantation sites were called “distal,” “center_1,” “center_2,” and “proximal,” in order from the distal position. The “distal” site was positioned 35 mm from the distal end of the femur, and the distance between each implantation site was 15 mm. Three types of implants (*ϕ*—4.5 mm) were tapped into drill holes manually. The location of each implant was randomly selected. Saline containing the antibiotic enrofloxacin (Bayer Yakuhin Ltd.) was used to avoid thermal necrosis. Immediately after implantation, radiographs were taken to confirm the implant placements. The animals were housed individually under a 12:12‐h light–dark cycle at a constant temperature and relative humidity and were allowed free access to food and water and unlimited exercise. For double staining of the bones, tetracycline yellow and calcein green were injected 9 and 2 days prior to bone collection, respectively. After 4, 8, and 12 weeks of implantation, the beagles were euthanized and the femurs were harvested. The extracted femurs were radiographed prior to analysis. The implants with bones at each site were cut with a band saw, and the implants at “center_1” and “proximal” were frozen at −80°C until mechanical testing was performed. For histological sections, the femurs at “distal” and “center_2” were immersed in 10% neutral buffered formalin (FUJIFILM Wako Pure Chemical) overnight, and then fixed with 70% ethanol.

### 2.3 Mechanical testing

Push‐out testing was performed using a universal testing machine (Model 5965, Instron, MA, USA). The femurs containing the implants (*n* = 4 in each implant) were defrosted and the soft tissues and cortex on the medial side were removed. The femurs were then fixed to a specially made jig using polymethyl methacrylate cement. A load at a cross‐head speed of 5 mm/min was applied, and the force‐displacement curve was recorded to determine the maximum push‐out force. The cortical thickness and implant diameter were measured after mechanical testing at six and three points using a digital caliper and micrometer, respectively, and the mean values were used. Interfacial shear strength was calculated by dividing the maximum push‐out force by the average interfacial area.

### 2.4 Histological evaluation

Implant‐containing femurs that were not assessed in the push‐out test were used for histological evaluation (*n* = 4 for each implant). A total of 36 implants were histologically evaluated. The implants with surrounding bone tissues were dehydrated in graded ethanol (70%–100%) and then embedded in methyl methacrylate resin. The embedded specimens were cut with a band saw perpendicular to the long axis of the femur and sectioned to 20 μm thickness using a microtome. Each section was stained with hematoxylin and eosin (H&E) and Villanueva Bone. H&E‐stained sections were used for histological evaluation and were observed under a light microscope (IX‐71; Olympus, Tokyo, Japan).

Quantitative histological analysis was performed using ImageJ software (National Institute of Health, MD, USA). The outer and inner layers of porous structures in cortical bones with dimensions of 2.0 × 0.5 mm were defined as the regions of interest (ROI). The ROI of inner layers was determined as area of 0.5 mm thickness from the end of inner layer, which corresponds to the single layer thickness. The ROI of outer layers were set adjacent to that of inner layers. The bone area (BA) and total area of the implant void (TA) in each ROI were measured. Bone area fractions (BA/TA) in each layer and the TA in the porous region (outer and inner layers) were calculated. A representative example of the analyzed image is shown in Figure 5A.

### 2.5 Statistical analysis

Quantitative data are expressed as mean ± standard error. EZR software^25^ (Saitama Medical Center, Jichi Medical University, Saitama, Japan) was used for statistical analysis. Bartlett's test was used to evaluate homoscedasticity. Nonparametric tests were applied when the variance was not homogenous (*p* < .05). For comparisons among the three implant types, Tukey's multiple comparison test and nonparametric Steel‐Dwass test were performed. The increase in interfacial shear strength and bone ingrowth with time were analyzed using Dunnett's test and the nonparametric Steel test for multiple comparisons over 4 weeks. *p* < .05 was considered statistically significant.

## 3 RESULTS

**FIGURE 2:**
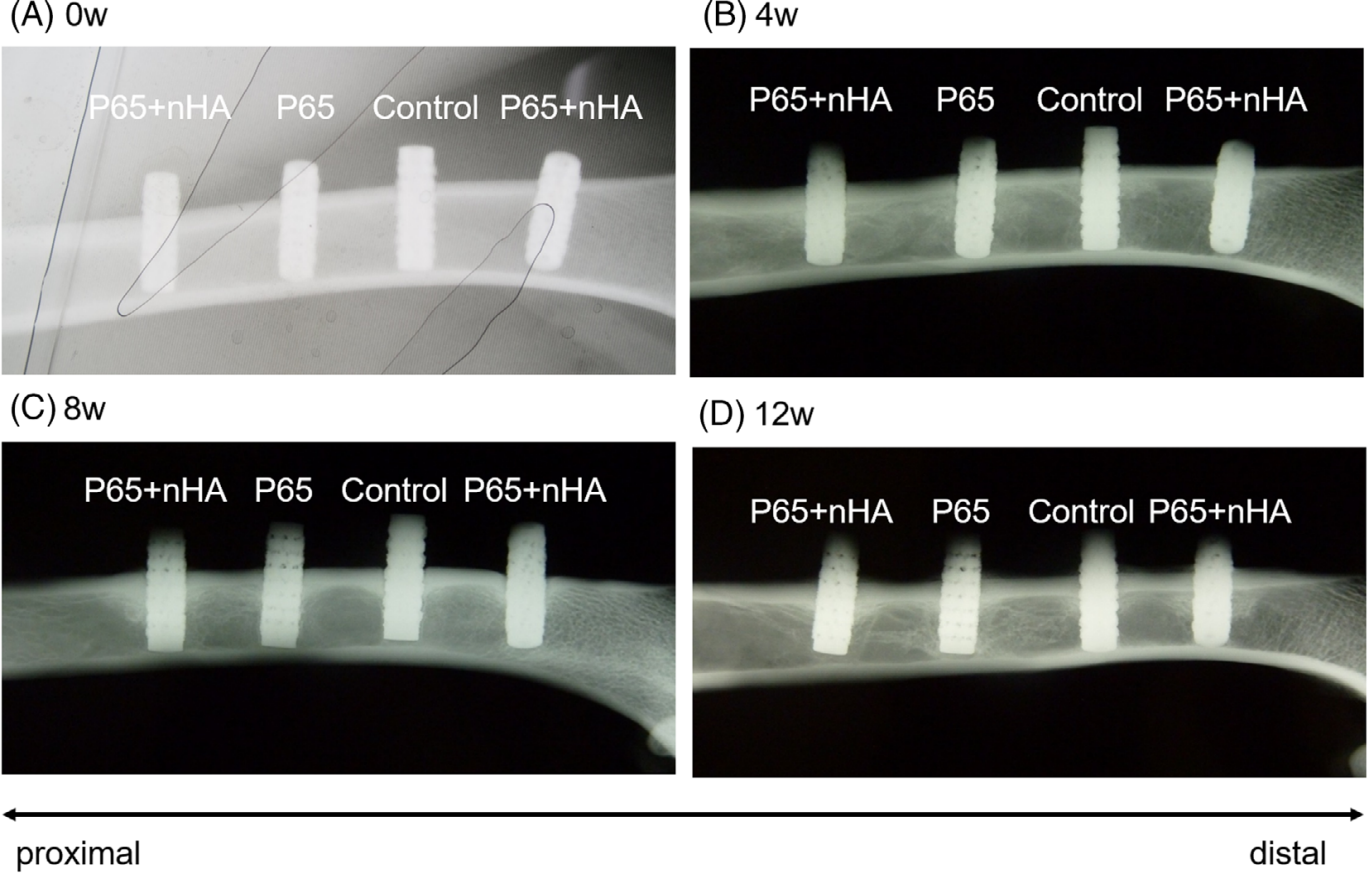
Radiographs of femurs harvested at 0, 4, 8, and 12 weeks postoperatively showing extensive peri‐implant bone formation around all implants

All animals recovered uneventfully after surgery and no infections or postoperative morbidities were observed. The body weights of the beagles did not change significantly during the animal experiment. Radiographs of explanted femurs demonstrated bone formation around the implants at all time points (Figure 2). Fluorescence labeling from Villanueva Bone‐stained sections confirmed new bone formation at all implantation periods (data not shown).

### 3.1 Mechanical testing

**FIGURE 3:**
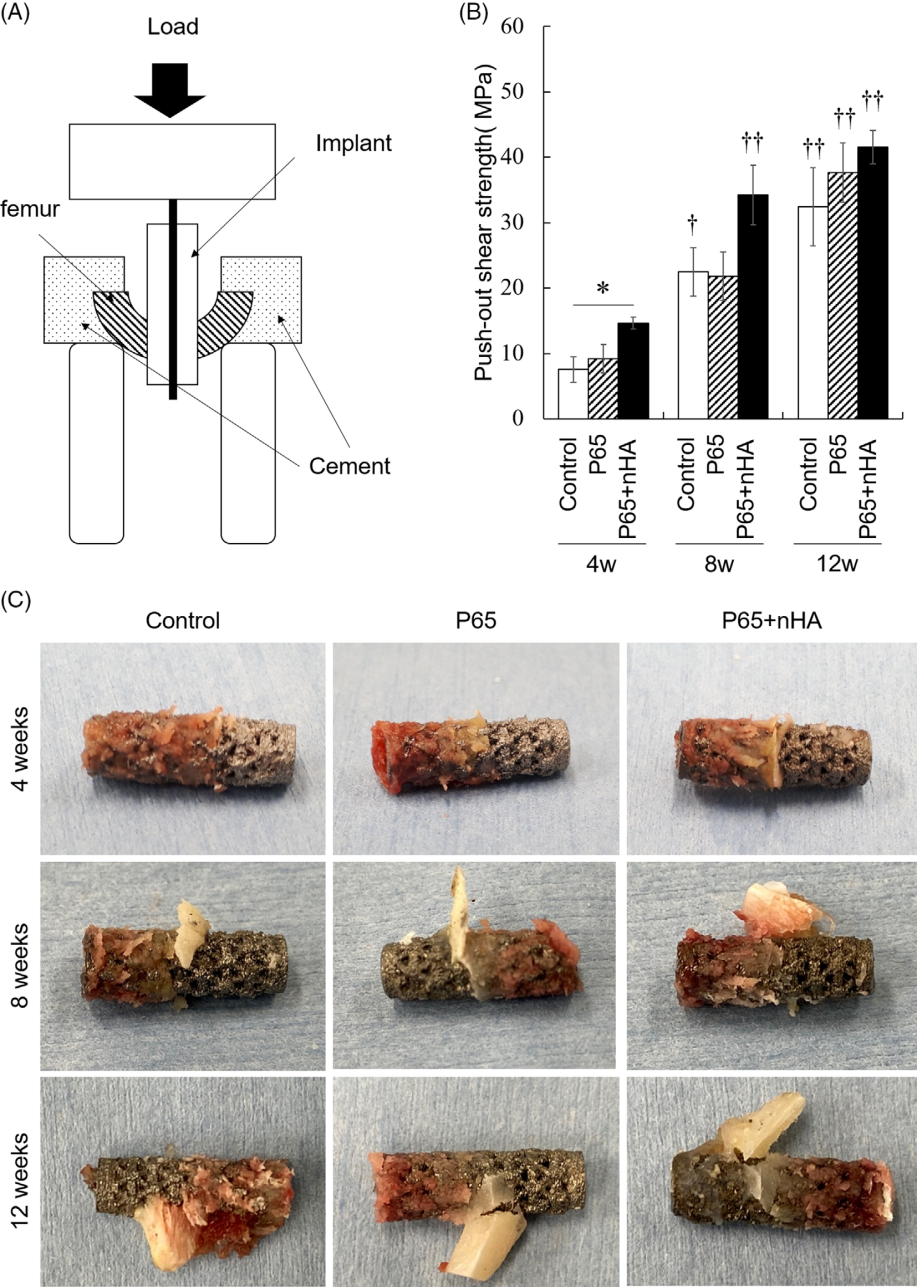
(A) Schematic illustration of the push‐out test. (B) Interfacial shear strength measured by push‐out test at 4, 8 and 12 weeks after implantation. (C) Pictures of the implants after the mechanical test. *p < .05 among implant types. †† indicate significant differences among implantation periods. †*p* < .05 and ††*p* < .01 versus 4 weeks

All implants at 4 and 8 weeks after implantation were successfully pushed out. However, two cortical bones tested at 12 weeks were fractured before implant removal (one had control implants and one had P65 + nHA) and thus, were not included in this study. Figure 3 shows the interfacial shear strength at 4, 8, and 12 weeks. The shear strength significantly increased from 4 to 12 weeks for all implants (control, *p* = .004; P65, *p* < .001; P65 + nHA, *p* < .001). At 4 weeks, P65 + nHA had significantly higher shear strength than control (*p* = .048), whereas there were no significant differences found between P65 and control (*p* = .787). P65 + nHA exhibited higher shear strength than P65 at 4 (*p* = .134) and 8 weeks (*p* = .127). At 12 weeks, all implants showed high shear strength. Images of the implants after the push‐out test are shown in Figure 3B. In P65 + nHA, abundant cortical bone apposition was observed around the implants compared to that in the control group and P65 at 4 weeks. The amount of bone apposition increased from 4 to 12 weeks in all implants.

### 3.2 Histological evaluation

**FIGURE 4:**
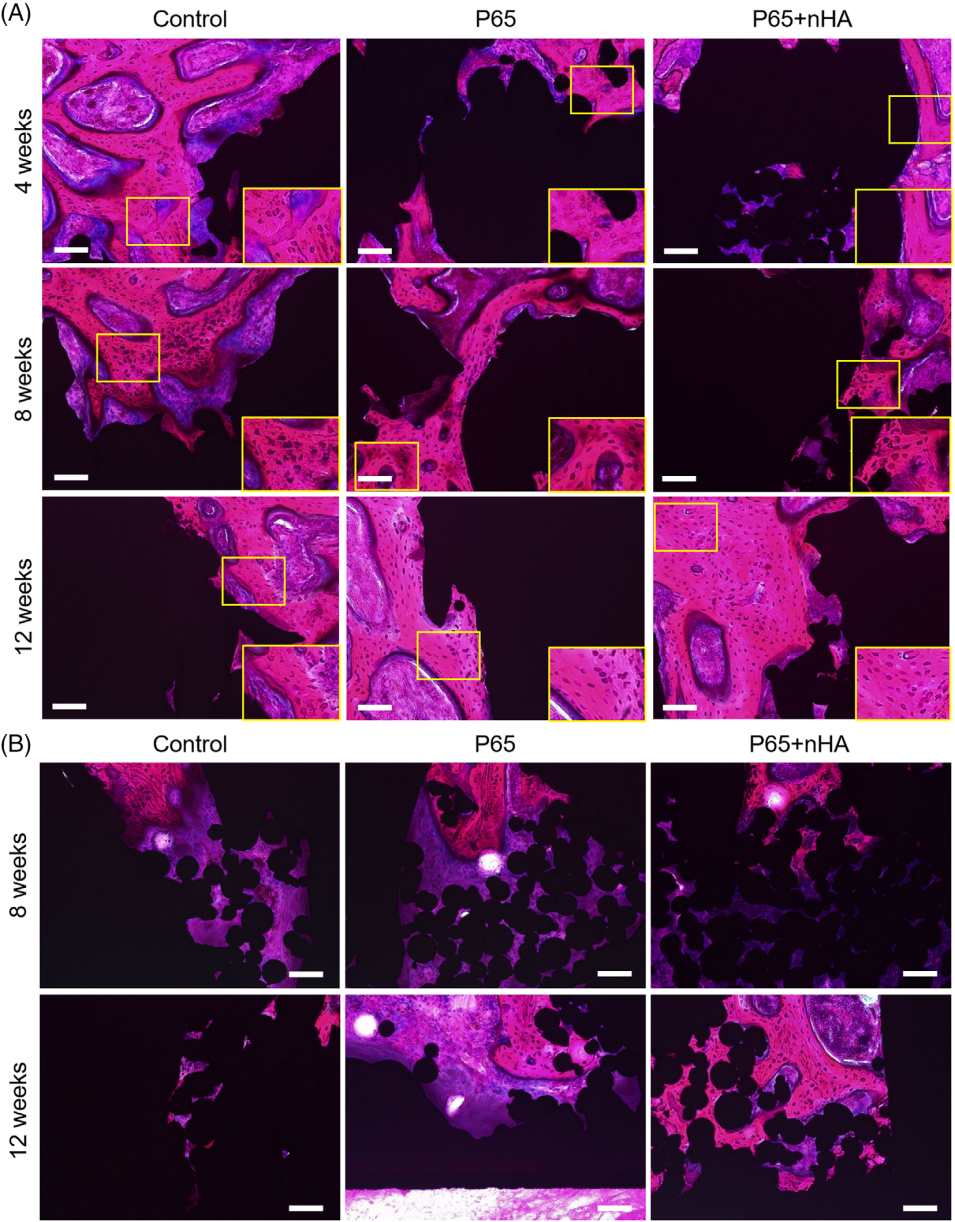
(A) Histological images of sections stained with hematoxylin and eosin at 4, 8, and 12 weeks. Scale bars = 100 μm. The insets show the magnified images of the yellow boxed area, revealing osteocytes in bone tissues. (B) Representative images of the inner layers at 8 and 12 weeks

Histological images of sections stained with hematoxylin and eosin are shown in Figure 4. At 4 weeks after implantation, a small number of osteocytes in the bone tissues were observed in all implants. A large number of osteocytes were observed at 8 and 12 weeks, indicating maturation of newly formed bones in the porous structures.

**FIGURE 5:**
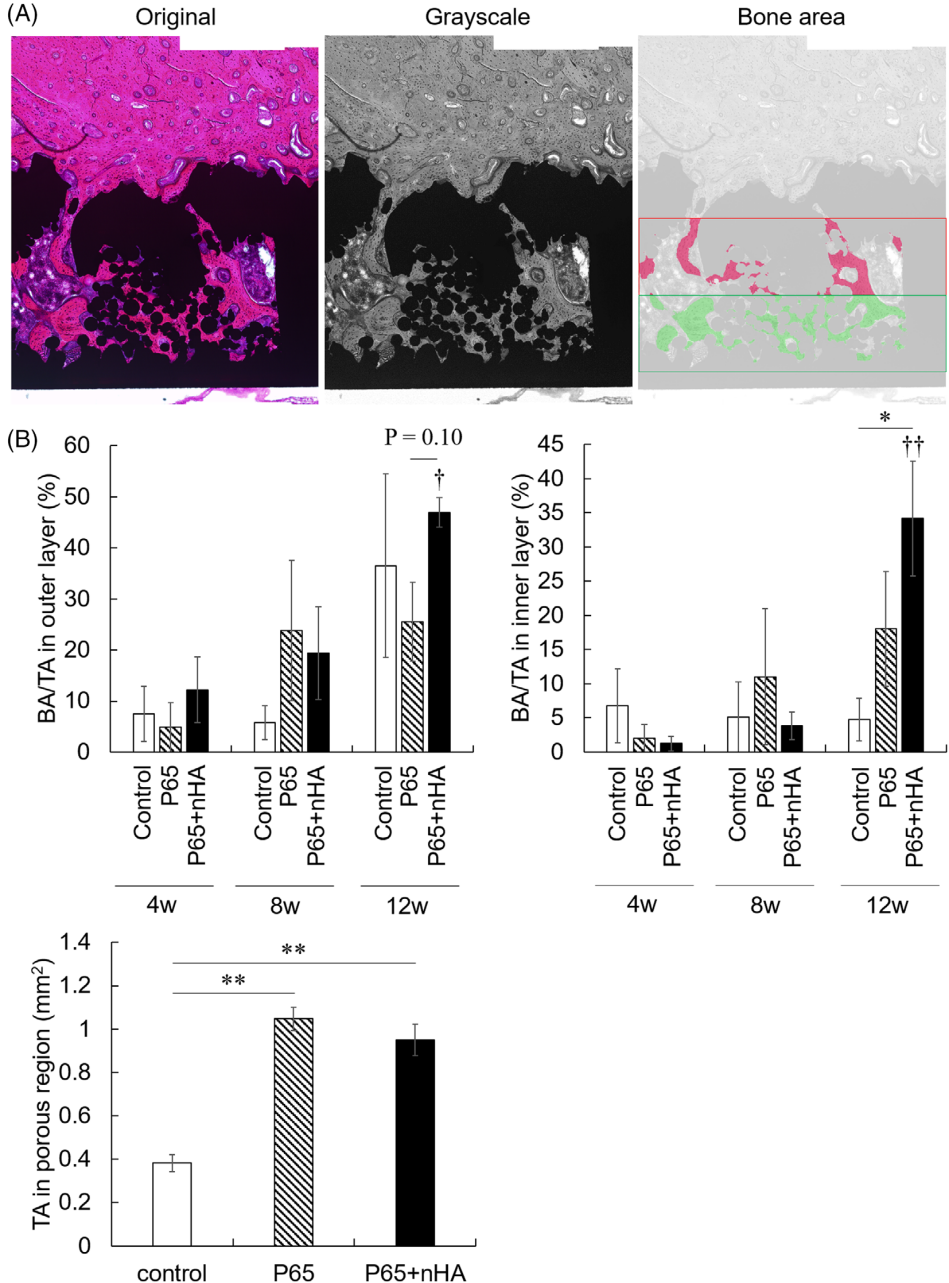
(A) Representative images used in bone ingrowth histological analysis. (B) Quantitative histological results of bone ingrowth and total area in the porous structures. *p < .05 and **p < .01 among implant types. †*p* < .05 and ††*p* < .01 versus 4 weeks

Figure 5 shows the quantitative results of histological analysis. P65 + nHA showed a significant increase in BA/TA in both layers from 4 to 12 weeks (outer layer: *p* = .020, inner layer: *p* = .038), whereas the control (outer layer: *p* = .491, inner layer: *p* = 0.934) and P65 (outer layer: *p* = .264, inner layer: *p* = .282) exhibited no significant increase. In the outer layer that was close to the host bone, no differences in BA/TA were observed among the three groups at 4 and 8 weeks; however, P65 + nHA showed a greater BA/TA in the outer layer at 12 weeks than P65 (*p* = .107). The BA/TA in the inner layer of P65 + nHA was higher than that of the control at 12 weeks (*p* = .040), but not that of P65 (4 weeks: *p* = .941, 8 weeks: *p* = .727, 12 weeks: *p* = .290). The BA/TA in P65 did not show an increase compared to that in the control group (4 weeks, *p* = .529; 8 weeks, *p* = .806; 12 weeks, *p* = .419). The sum of the TA at 4, 8, and 12 weeks for each implant was calculated. The TA significantly increased in P65 and P65 + nHA compared to that of the control (*p* < .001), but there were no significant differences between P65 and P65 + nHA (*p* = .447).

## 4 DISCUSSION

It is increasingly recognized that the structural characteristics of the implant surface are important determinants of functional bone tissue regeneration.^26^, ^27^ In the present study, we investigated the mechanical stability and bone responses around nHA‐coated EBMed 3D porous orthopedic implants. Our findings show that our novel nHA coating successfully improved mechanical fixation and bone ingrowth.

Nano‐sized hydroxyapatite coating has been used on implant materials such as polyether ether ketone and titanium^21^, ^22^, ^28^ using electrochemical techniques or dipping and heat treatment processes. Unlike these coatings, our hydroxyapatite nanoparticles were covalently coated on the implant surface by a silane coupling agent to keep the strong linkage of nHA with implants surface, even in deep regions of the 3D porous structures. The quantitative analysis of linkage strength between nHA and implant will be reported in our future study. In addition, our novel coating technique did not use high‐temperature treatments, which could degrade the mechanical properties of Ti‐6Al‐4V implants.

The porosity of the EBMed 3D porous implants used in this study was qualitatively evaluated using histological sections (Figure 5). The total area of the implant void corresponds to the porosity of the implant surfaces. There were no significant differences in post‐implantation TA observed between P65 and P65 + nHA, indicating that nHA did not clog the pores of the EBMed 3D porous implant. TA comparisons indicated that the porosities of the P65 porous structures (P65 and P65 + nHA) were greater than those of the control. FE‐SEM images confirmed the nHA coating on the EBMed 3D porous implants. These results suggest that nHA is a suitable coating material for 3D porous Ti‐6Al‐4V implants fabricated using additive manufacturing techniques.

The mechanical stability of implants is an important factor for secondary bone ingrowth. Micromotion at the implant‐bone interface prevents bone ingrowth due to the induction of fibrous tissue growth^29^, ^30^ and may result in the occurrence of radiolucent lines around the acetabular components. Therefore, better initial bone‐implant fixation is needed to reduce radiographic signs. Our results demonstrated an increased initial (4 weeks) implant fixation by the combination of higher porosity and novel nHA coating, but increased porosity alone had no effect on bone‐implant fixation (Figure 3). The interfacial shear strength is affected by the friction coefficient^31^ as well as osseointegration. The spikes and outer layer of the EBMed 3D porous implants, which were adjacent to host bone tissues, were the same, indicating that the friction coefficient was the same among the three implants. Abundant fusion of cortical bone was confirmed in nHA‐coated implants at 4 weeks compared to the other implants. Additionally, the mechanical properties of the bone closest to the implants may also influence the interfacial shear strength. The elastic modulus and hardness of bone tissues around nHA‐coated implants have been reported to be higher than those around non‐coated titanium implant.^32^ Overall, newly formed bone tissues with improved mechanical properties owing to the novel nHA coating possibly contributed to better initial implant fixation.

This study proposes a novel nHA coating as a strong mediator to stimulate bone growth into complex 3D structures fabricated by additive manufacturing. Histological analysis demonstrated the combined effect of higher porosity and the novel nHA coating on bone ingrowth. Increased porosity alone did not increase bone ingrowth to the deep region of the porous structure, but the novel nHA coating on implants significantly improved bone ingrowth compared to the low‐porosity implant. The permeability of the structure, which is defined by the Wang and Tarbell's equation,^33^ affects cellular activities and bone growth into a porous implant. Low permeability inhibits the inflow of blood, and subsequently results in poor cell penetration, osteogenic differentiation and bone ingrowth.^34^ We showed that the porosities were improved in the P65 and P65 + nHA implants, indicating increased permeability of the porous structures. In addition, coating biomimetic HA on Ti‐6Al‐4V implants has been reported to enhance bone ingrowth depth.^35^ Therefore, using a high porosity implant with a novel nHA coating had a combined advantageous effect on the ingrowth depth (total length from the outer to inner layers) of bone tissue; thus, its surface could provide high bioactivity. Additionally, the novel nHA coating continued the progression of bone ingrowth from 4 to 12 weeks, while non‐coated EBMed 3D porous implants (control, P65) did not significantly increase bone ingrowth progression. Although the osteoconductive mechanism of nHA coatings remains unclear, various studies have reported improved gene expression in nHA‐coated implants. In a rabbit tibia model, nanostructured calcium phosphate‐coated implants were associated with upregulated expression of osteogenic genes, such as alkaline phosphate (ALP) and osteocalcin.^36^ nHA coating also improves gene expression (runt‐related transcription factor 2, ALP, and osteopontin) and bone formation in both healthy and diabetic rat tibias.^37^ Therefore, nHA coating may act as an osteogenic scaffold that stimulates osteoblastic activity over a long period of time.

Bioactive coatings that induce greater bone formation have been clinically reported to have a positive effect on radiographic signs around porous acetabular cups.^38^, ^39^ In over 70% of cases, a gap between the host bone and the alkali‐ and heat‐treated titanium acetabular cups was observed by radiographic evaluation immediately after surgery; however, all the gaps disappeared within a year. In HA‐coated acetabular cups, radiolucent lines observed at 1 month follow‐up decrease after 1 year, whereas the highly porous titanium cups remained radiolucent. Our findings indicate that the novel nHA coating has the potential to prevent long‐term radiolucent lines around 3D porous Ti‐6Al‐4V implants. To the best of our knowledge, the present study is the first to describe the mechanical and biological characterization of nHA‐coated 3D porous Ti‐6Al‐4V implants additively manufactured via EBM.

The present study had some limitations. First, the use of a beagle transcortical model to assess the mechanical and biological responses of the nHA‐coated EBMed implant does not adequately reflect clinical situations. A transcortical model is generally used for the initial characterization of new porous implants.^4^, ^40^ However, radiolucent lines around acetabular cups occur in cortical and cancellous bones. Further studies are necessary to demonstrate the response to nHA‐coated implants in cancellous bone. The advantage of nHA coating on preventing the occurrence of radiolucent lines should be clarified more precisely by comparing the implants including negative control group and will be reported in our future study. Second, the measured interfacial shear strength at 8 and 12 weeks probably represents a lower value because some of the surrounding cortical bones were integrated with the implants after the push‐out test. Although P65 + nHA exhibited better bone ingrowth ability than the control at 12 weeks, the bone‐implant shear strength did not increase. Modification of animal species, implant location, and implant sizes are required to evaluate the precise mechanical stability of implants. Third, manual insertion of implants into the drill holes might have caused differences in the press‐fit conditions of each implant.

## 5 CONCLUSION

We assessed the mechanical stability and bone ingrowth of a newly developed 3D porous implant coated with nHA. This porous implant has high bioactivity, which induced greater initial stability and deeper bone ingrowth than low‐porosity EBMed implant. The findings of this study can provide new surface modification strategies for EBMed orthopedic implants and contribute to the prevention of radiolucent lines.

## CONFLICT OF INTEREST

Ryota Watanabe and Hiroyuki Takahashi are employees of Teijin Nakashima Medical, a manufacturer of orthopedic implants. The other authors declare that they have no conflicts of interest.

## Data Availability

The data of our findings are available from the corresponding author upon reasonable request.
